# Posterior reversible encephalopathy syndrome in a patient with mixed connective tissue disease: a case report

**DOI:** 10.1186/s13256-016-0955-y

**Published:** 2016-06-02

**Authors:** Reza Rahmanzadeh, Ramin Rahmanzade, Mozhdeh Zabihiyeganeh

**Affiliations:** 1 Division of Neuroscience, Cellular and Molecular Research Center, Iran University of Medical Sciences, Tehran, Iran; 2 Division of Neuroscience, Neurology Research Center, Shahid Beheshti University of Medical Sciences, Tehran, Iran; 3 Bone and Joint Reconstruction Research Center, Shafa Orthopedic Hospital, Iran University of Medical Sciences, Tehran, Iran

**Keywords:** Posterior reversible encephalopathy syndrome, Mixed connective tissue disease, Cyclophosphamide

## Abstract

**Background:**

Posterior reversible encephalopathy is a syndrome highly associated with hypertension and cytotoxic therapy. The syndrome typically presents with headache, visual abnormality, seizures and characteristic vasogenic edema on magnetic resonance imaging. The entity warrants a prompt diagnosis to avoid deteriorating consequences.

**Case presentation:**

In this report, we describe a 15-year-old Iranian boy who was diagnosed with mixed connective tissue disease, and cyclophosphamide pulse therapy was administered. Three days after the second pulse of cyclophosphamide, when he was receiving prednisolone and hydroxycholoroquine, our patient developed generalized tonic-clonic seizures. Magnetic resonance imaging findings showed high signal intensities in the posterior areas of his brain. After 8 days, the brain magnetic resonance imaging abnormalities were resolved following the control of his blood pressure and antiepileptic treatment. These observations have been indicative of posterior reversible encephalopathy syndrome. Nevertheless, our patient developed uncontrollable respiratory distress and eventually died.

**Conclusions:**

To the best of our knowledge, this case is the first report of posterior reversible encephalopathy syndrome in a patient with mixed connective tissue disease. As the patient developed posterior reversible encephalopathy syndrome 3 days after cyclophosphamide pulse therapy to reduce the disease activity, it is hard to accurately determine whether posterior reversible encephalopathy syndrome in this case is a complication of cyclophosphamide or a condition that resulted from the mixed connective tissue disease flare-up.

## Background

Posterior reversible encephalopathy syndrome (PRES) is a life-threatening condition which can be characterized by symmetric involvement of posterior white matter on magnetic resonance imaging (MRI) and neurological impairments such as seizures, altered mental status, headache, and visual disturbances [1, 2].

PRES has been reported in different conditions such as hypertensive encephalopathy, eclampsia, thrombotic thrombocytopenia purpura, and rheumatologic disorders [3–5].

The mainstay of management of PRES is timely diagnosis and discontinuation of causative agents that may prevent subsequent abnormalities of the central nervous system.

The extensive use of immunosuppressive therapy and the autoimmune nature of rheumatologic diseases may make patients more vulnerable for developing PRES in the course of disease. Nevertheless, to the best of our knowledge, PRES has not been reported as a complication of treatment or a manifestation of disease in patients with mixed connective tissue disease (MCTD).

In this report, we describe a 15-year-old Iranian boy with MCTD who presented with PRES 3 days after cyclophosphamide pulse therapy when he was receiving a high dose of steroids. Our patient was treated with antihypertension and antiepileptic medications and a repeat MRI scan showed no abnormality 8 days later.

## Case presentation

**Fig. 1:**
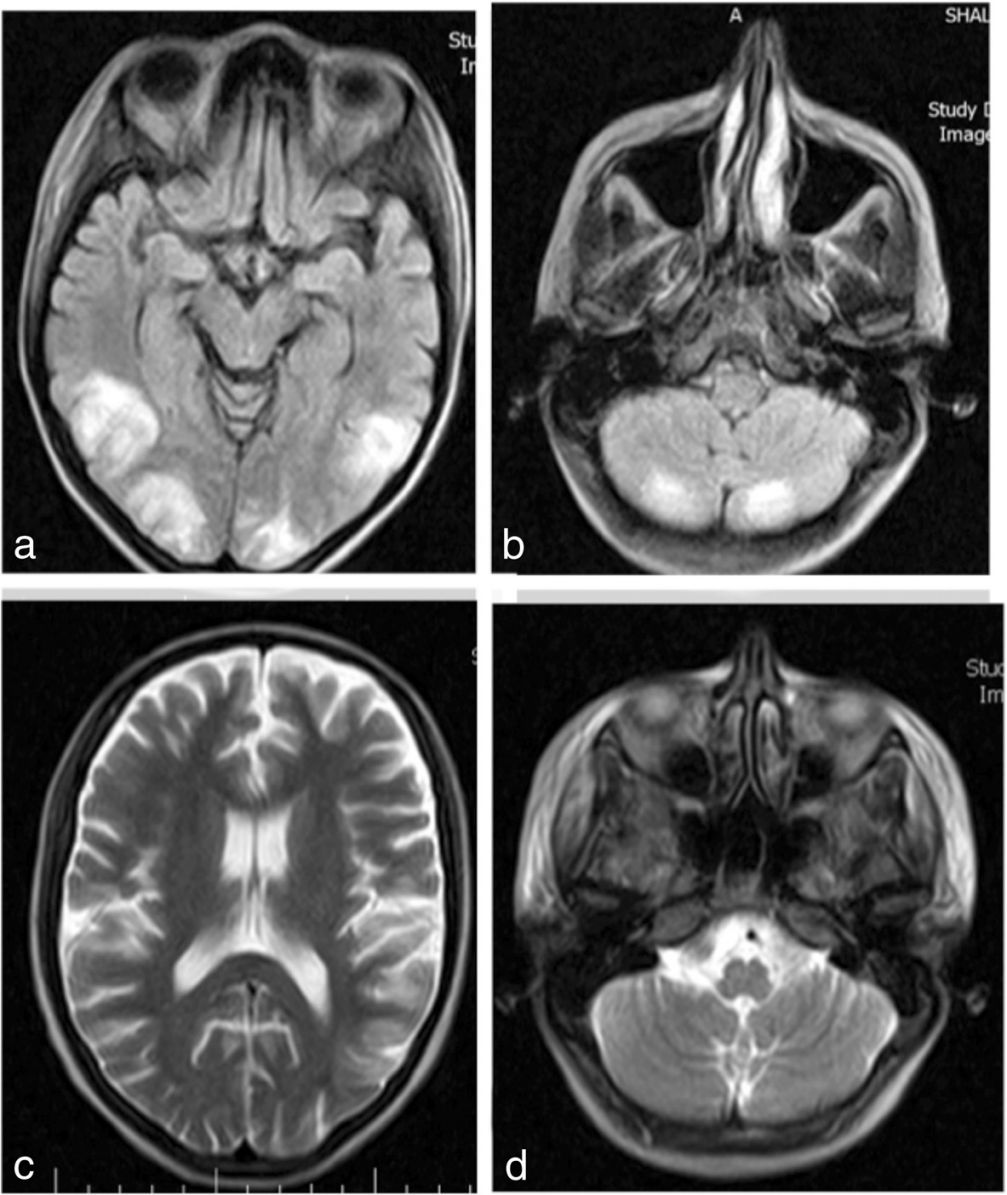
Brain magnetic resonance imaging T2-weighted/fluid-attenuated inversion recovery scan showing high signal intensities in **a** the subcortical white matter of occipital, posterior parietal, and posterior temporal lobes and **b** the cerebellum. **c**, **d** Follow-up brain magnetic resonance imaging T2-weighted/fluid-attenuated inversion recovery scan 8 days after the first imaging showed complete resolution

Our patient was a 15-year-old Iranian boy with a 2-year history of skin ulcer compatible to pyoderma gangrenosum. From the onset of his skin problems, he had been receiving a low dose of steroids, which was increased to 1 mg/kg 2 months prior to admission. He was referred to our hospital following development of muscle weakness and severe dyspnea. History-taking revealed a 1-year history of discoloration of his fingers in cold temperatures. A physical examination showed scleroderma-like signs of acrosclerosis and a small mouth orifice with difficulty in opening. Blood tests showed a remarkable elevation of muscle enzymes (creatine phosphokinase [CPK] >3000, aldolase 39.4 and lactate dehydrogenase [LDH] 1510) and electromyogram-nerve conduction (EMG-NCV) tests indicated chronic moderate to severe myopathic process. We performed a muscle biopsy of his left deltoid muscle that revealed multiple necrotic fibers and extensive inflammatory endomysial foci. The laboratory findings showed antinuclear antibodies (ANA) 1:2500 positive, anti-double-stranded (ds) DNA 198 positive, anti-SM >200 positive, anti-SCL-70 >200 positive, anti-centromere >2 positive, anti-U1 RNP 178.4 positive, white blood cells (WBC) (from 3500 to 6900 during the hospitalization), hemoglobin (Hb) 12.3, platelets (PLT) 128,000, and erythrocyte sedimentation rate (ESR) 56 (normal range <30). His anticardiolipin, anti-beta 2 glycoprotein I and lupus anticoagulant antibodies were negative and also his complement 3 (C3) and complement 4 (C4) levels were in normal range. Our patient fulfilled the Alarcon-Segovia diagnostic criteria [6] with positive serology and three of the five clinical criteria especially Raynaud’s phenomenon, acrosclerosis, and myositis. Our patient also met the Kasukawa diagnostic criteria [6] with one common symptom of Raynaud's, positive serology, and mixed findings of leukopenia/thrombocytopenia, acrosclerosis, and muscle weakness. A chest X-ray showed diffuse pulmonary infiltration and a computed tomography (CT) scan reported a bronchiolitis obliterans organizing pneumonia (BOOP) reaction. Further tests also showed heart failure (ejection fraction [EF] = 30%) and pulmonary arterial hypertension (pulmonary artery pressure [PAP] = 75 mmHg). Pulmonary embolus was ruled out by CT angiography. According to the criteria, our patient was diagnosed with mixed connective tissue disorder (MCTD), and 1 g intravenous methylprednisolone was administered. Then our patient received 500 mg cyclophosphamide pulse therapy, and was discharged with prednisolone 70 mg daily and hydroxycholoroquine 200 mg daily. Our patient received the second pulse of 500 mg cyclophosphamide 2 weeks later. Three days after the second cyclophosphamide pulse when he was receiving prednisolone 70 mg/day, he developed several generalized tonic-colonic seizures. After admission to the intensive care unit, he developed another seizure that lasts 3 minutes. At this time, his blood pressure was 170/130. Therefore, phenytoin and antihypertension drugs were prescribed. An MRI scan of our patient revealed high signal intensities on T2-weighted images and fluid-attenuated inversion recovery (FLAIR) sequences in the subcortical white matter of the occipital, posterior parietal, and posterior temporal lobes, and the cerebellum (Fig. 1). After 8 days, the brain MRI abnormalities had completely been resolved. These observations have been indicative of PRES.

From the first day of his second admission, our patient presented fever, cough and dyspnea and the laboratory tests showed creatinine 2.35 mg/dL, urea 182 mg/dL, WBC 3500, and platelets 75,000. A chest X-ray revealed extensive pleural effusion. In a peripheral blood smear, there was no evidence of thrombotic thrombocytopenia purpura. The serial blood test showed a progressive increase in his creatinine level and decrease in his platelet count. Therefore, we started rituximab 500 mg for 2 weeks to control the disease flare-up. However, rituximab was not efficient and we started intravenous immunoglobulin (IV Ig) for 5 days. IV Ig did not improve our patient and we had to initiate plasma exchange. However, his creatinine level increased and respiratory symptoms became worse. Dialysis prevented the worsening of his condition and relieved our patient’s symptoms. The results from bronchoalveolar lavage (BAL) showed infection to cytomegalovirus (CMV), Gram-negative bacillus, and candida. Although our patient received broad-spectrum antibiotics and antifungal agents, his respiratory manifestations were not improved and he was intubated. After 5 days of intubation, our patient developed heart arrest and, following 45 minutes of cardiopulmonary resuscitation (CRP), our patient died.

## Discussion

Posterior reversible encephalopathy syndrome was first introduced by Hinchey and his colleagues in a study of 15 patients [7].

PRES can be diagnosed in a patient with reversible neurological manifestations including headache, nausea/vomiting, visual abnormalities, consciousness impairment, seizure activity, and focal neurologic signs, in conjunction with bilateral involvement of posterior brain areas on magnetic resonance imaging [2].

The pathophysiology of PRES remains elusive. However, it has been suggested that a compromised cerebrovascular autoregulation due to acute hypertension may play a pivotal role. Accordingly, impaired cerebrovascular regulation may lead to arteriole leakage and cerebral vasogenic edema [1, 2]. Although the co-occurrence of hypertension and PRES is remarkable, other possible mechanisms have also been reported to be important in PRES such as disrupted cerebral autoregulatory mechanisms due to autonomic dysfunction [8], breakdown of blood-brain barrier following cytotoxic agents-induced endothelial toxicity, autoimmunity, and sepsis [9].

In addition, immunosuppressive agents such as methylprednisolone, dexamethasone, cyclosporine and cyclophosphamide have been reported to be related to PRES [10].

Our patient presented PRES 3 days after cyclophosphamide pulse therapy when he was receiving a high dose of prednisolone in the setting of a MCTD flare-up. On the other hand, clinical signs showed that our patient developed sepsis concomitant with PRES. Therefore, we could not accurately determine the main causative source of PRES among the cyclophosphamide pulse therapy, prednisolone, sepsis, and the flare-up of the underlying disease. Our patient had a 2-year history of taking steroids for his dermatological disorder that may decrease the importance of maintenance prednisolone as the cause of PRES. Most of the reported cases of PRES are suggested to be due to cytotoxic or steroid therapy. In the present case, the diagnosis of PRES a few days following the second pulse of cyclophosphamide may underscore its offensive effects and may suggest the cyclophosphamide pulse as a potential cause. Nevertheless, several reports show presentation of PRES unattributable to therapy in patients with rheumatologic diseases, which suggested it to be a condition resulting from underlying disease and even, in some cases, PRES was the first manifestation of the disease [11]. As in our case, PRES was concomitant with sepsis, therefore sepsis may play a role in this condition.

From the standpoint of pathogenesis, MCTD may be strongly suggestive as a cause of PRES. Various studies highlight that endothelial cell damage resulted from different autoantibodies in MCTD [12]. Along these lines, vasculopathy has been suggested to be a specific feature of MCTD that may lead to pulmonary arterial hypertension, which is responsible for most of the deaths in the late stages of MCTD [13]. In addition, the dysfunction of the autonomic system has been shown in MCTD [14]. Therefore, PRES may be a manifestation of MCTD in our case because our patient presented PRES during the disease flare-up.

To the best of our knowledge, a case of PRES in a patient with mixed connective tissue disease has not yet been reported. The neurological manifestations of MCTD have been believed to be less frequent than findings of other systems. Although the main neurological manifestations of MCTD are trigeminal neuropathy, headaches, and aseptic meningitis, this report suggests PRES as a neurological condition which may occur during the course of MCTD [15]. Although there is no difference between our patient and previously reported cases in terms of PRES characteristics, further reports are needed for better understanding of PRES in MCTD.

## Conclusions

To the best of our knowledge, this case is the first report of posterior reversible encephalopathy syndrome in a patient with mixed connective tissue disease. As the patient developed PRES shortly after the cyclophosphamide pulse, during an MCTD flare-up, and concomitant with sepsis, it is hard to determine the accurate cause of PRES in this case. Taken together, MCTD may be strongly suggestive as the cause of PRES for the extensive endothelial dysfunction involved in its pathogenesis.

## Consent

Written informed consent was obtained from the patient’s family for publication of this case and any accompanying images. A copy of the written consent is available for review by the Editor-in-Chief of this journal.

## Abbreviations

CT, computed tomography; MCTD, mixed connective tissue disease; MRI, magnetic resonance imaging; PRES, posterior reversible encephalopathy syndrome.
